# A Parametric Design Method for Optimal Quick Diagnostic Software

**DOI:** 10.3390/s19040910

**Published:** 2019-02-21

**Authors:** Xiao-jian Yi, Peng Hou

**Affiliations:** 1 School of Mechatronical Engineering, Beijing Institute of Technology, Beijing 100081, China; yixiaojian@amss.ac.cn; 2 Academy of Mathematics and Systems Science, Chinese Academy of Sciences, Beijing 100190, China; 3 Department of Overall Technology, China North Vehicle Research Institute, Beijing 100072, China

**Keywords:** early diagnosis, time-critical application, early classification, feature selection

## Abstract

Fault diagnostic software is required to respond to faults as early as possible in time-critical applications. However, the existing methods based on early diagnosis are not adequate. First, there is no common standard to quantify the response time of a fault diagnostic software to the fault. Second, none of these methods take into account how the objective to improve the response time may affect the accuracy of the designed fault diagnostic software. In this work, a measure of the response time is provided, which was formulated using the time complexity of the algorithm and the signal acquisition time. Model optimization was built into the designed method. Its objective was to minimize the response time. The constraint of the method is to guarantee diagnostic accuracy to no less than the required accuracy. An improved feature selection method was used to solve the optimization modeling. After that, the design parameter of the optimal quick diagnostic software was obtained. Finally, the parametric design method was evaluated with two sets of experiments based on real-world bearing vibration data. The results demonstrated that optimal quick diagnostic software with a pre-defined accuracy could be obtained through the parametric design method.

## 1. Introduction

In time-critical applications, fault diagnostic software is required to respond to a fault as early as possible. Based on the timely diagnostic result, operators can prevent the aggravation of consequences over time, such as the risks associated with crew member exposure to toxic or dangerous chemicals [1], the wrong operation of medical robots [2], error in numerical control processing technology [3], and fracture and crack arrest in ship structures [4].

To achieve this aim, many methods have been applied to enable diagnostic software to respond to faults as early as possible. Those methods are divided into two categories. The first is based on signal processing technologies such as wavelet transform [5,6], empirical mode decomposition (EMD) [7], spectral kurtosis (SK) [8], envelope spectrum [9,10], and others [11,12,13]. These methods assume that the fault feature changes from weak to strong with the development of the fault. By extracting the weak feature of faults in the frequency domain or the time–frequency domain, a diagnostic result can be obtained in the early stage of fault development, and the objective of receiving a response as early as possible is achieved. Due to the weak nature of early fault features, weak fault features are often submerged in the noisy background, which increases the difficulty of feature recognition. To address this challenge, those methods apply noise-reduction methods before the recognition of weak features [14]. Although useful weak features are extracted, the feature information is also weakened in the process of noise reduction [15], which means that the performance of the fault diagnostic software is degraded. To avoid this situation, the stochastic resonance (SR) method was applied [16,17,18,19]. However, there are still some difficulties in practical applications. Firstly, the difficulty of feature recognition is not reduced by the noise-reduction method and stochastic resonance (SR) method. Secondly, even if the noise is reduced successfully, it is hard to know what the corresponding weak fault features are in the early stage of faults without enough knowledge of the fault mechanism. Additionally, the aforementioned methods cannot provide an a priori estimate on the diagnostic accuracy. Fortunately, through the combination of statistical analysis and signal processing technologies, statistical spectral analysis has been recently employed to improve this situation [20,21].

Another effective way is to increase the response rate of the diagnostic software by shortening the collection time of signals. Essentially, this kind of method speeds up the process of fault diagnosis in the software. In these methods, time series classification in machine learning is applied to shorten the collection time of signals [22,23,24,25]. As such, the fault diagnostic software is actually a classifier, which is trained to classify on as short as possible a prefix of the specified signal size. When the trained classifier is deployed in service, there is no need to wait for the whole specified signal size to be collected. Therefore, the diagnostic software can respond to the fault quickly. Compared with the approach based on signal processing technologies, these methods focus on the differences between the normal signal and the fault signal that emerge in the time domain. They assume that part of the signal required by the diagnostic software is redundant or useless. In addition, they need no knowledge of fault mechanism for extracting fault features. However, the application of these methods is still limited due to the insufficient data available for training the classifier.

As stated previously, although many approaches have been proposed to obtain a response as early as possible, to the best of our knowledge, there is no widely available quantified measure to assess whether the response time is improved by such methods. These approaches assume that the response time obtained by the software can be objectively evaluated without taking into account the effect caused by the hardware. Furthermore, it is assumed that the response time can be quantitatively assessed, which means different approaches can be evaluated against a common standard, and a reference can be provided in software design. On the other hand, a design to respond to faults as early as possible may be achieved at the expense of the accuracy of the fault diagnostic software. As mentioned in [26], a fault diagnostic system designed to respond as early as possible is very sensitive to noise. This seriously weakens the fault tolerance of the fault diagnostic system. It follows that the requirement for fault diagnostic software to respond as early as possible may lead to low accuracy. Therefore, there is a trade-off between the objective response time and the accuracy. In fact, it is pointless to require fault diagnostic software to respond as early as possible without considering the accuracy of its response. On the other hand, if the fault diagnostic software is only required to diagnose accurately without consideration for the response speed, the diagnostic result is more likely to be a “death certificate” [27]. These challenges motivated us to present a measure of the response time and to provide a novel design method based on a machine learning method to design an optimal diagnostic software based on this measure.

The contribution of this paper is two-fold:

(1) In order to carry out the parametric design of an optimal quick diagnostic software for time-critical applications, a measure of the response time is proposed. By combining the time complexity of the algorithm with the calculation formula of the signal acquisition time, we can quantitatively and rationally evaluate whether the response time is improved.
(2) We present a parametric design method for the optimal quick diagnostic software. This method adopts time series classification in machine learning as the diagnostic algorithm. The objective is to minimize the measure of the response time, and the constraint is to guarantee that the accuracy of the diagnostic software is no less than a pre-defined accuracy. An improved wrapper method is used to solve the optimization model in order to acquire the design parameters.

The remainder of the current paper is organized as follows. Section 1 elaborates the measure of the response time, which is adopted in our proposed design method. Section 2 details the steps in the parametric design method. Section 3 illustrates the application of the parametric design method through two experiments. In Section 4, the experimental results are detailed along with discussions. Finally, Section 5 concludes this paper.

## 2. The Measure of the Response Time

The response time is actually the time lag Δt between the time when the fault occurs and the time when the fault diagnostic software responds. It is mainly composed of the signal acquisition time and the execution time of the diagnostic algorithm in the diagnostic software. The signal acquisition time $t_{A}$ is actually the waiting time for the diagnostic software to acquire a specified signal size (the transmission delay is small enough to be negligible). The specified size for the input signal is related to the methods used in the diagnostic algorithm. In this way, enough signal information can be acquired by the diagnostic algorithm, rendering it able to execute fault diagnosis. Theoretically, the waiting time can be represented by $t_{A}=\frac{n}{f}$. The sampling number n quantifies the signal size. In most cases, the sampling frequency f is given ahead of most design parameters, thus the sampling number n is the only factor that determines $t_{A}$.

The time of the diagnostic algorithm execution $t_{B}$ can be estimated by the time complexity. This is generally expressed as a function of the size of the input [28]. Using the time complexity, the execution time $t_{B}$ can be represented as $t_{B}=O\left(m\right)$, where m is the size of the algorithm input (the size of the input signal). Equivalently, the size of the algorithm input can be replaced by the sampling number n for the fault diagnostic algorithm, thus the expression can be represented by $t_{B}=O\left[C\left(n\right)\right]$, where C is related to the type of diagnostic algorithm. In general, for the different types of algorithms, C(n) typically includes $n,\;n*logn,\;n^{\alpha }$, and $2^{n}$. Therefore, the execution time of the diagnostic algorithm $t_{B}$ is determined by the sampling number n and the algorithm type C.

**Figure 1:**
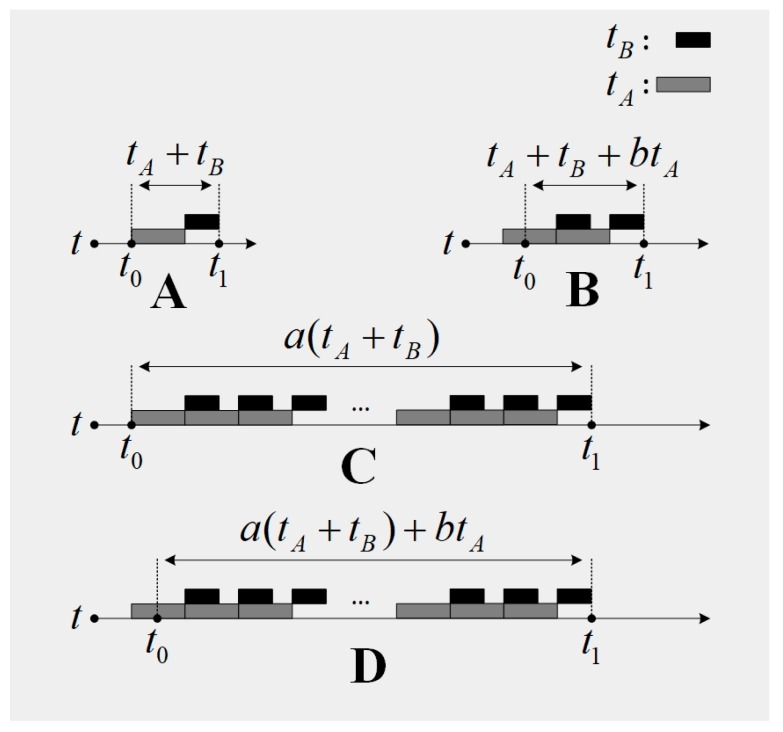
The constitution of the time lag Δt. (**A**,**B**) illustrate the situation that the diagnostic software has enough robustness. (**C**,**D**) illustrate the situation that the diagnostic software has low robustness.

When a diagnostic software is in service, the time lag Δt can be represented as $\Delta t=t_{1}-t_{0}\approx a\left(t_{A}+t_{B}\right)+bt_{A}$, where $a\in N^{*}$, b∈[0,1). $t_{0}$ is the time when the fault occurs, and $t_{1}$ is the response time of the fault diagnostic software. In our approach, a denotes the number of loops. A loop is a diagnostic procedure which is composed of the signal acquisition and the diagnostic algorithm execution. Due to the low robustness of the diagnostic software, the software should respond to the fault when there is severe interference or some sort of fault in the fault diagnostic software. If this situation takes place, the quantity a may not be 1 (as is described in Figure 1C,D). Generally, the diagnostic software in time-critical applications is assumed to have enough robustness, therefore the quantity a is set to be 1 in our approach (as is described in Figure 1A,B). The quantity b describes the point when the fault occurs in $t_{A}$. Its contribution is to guarantee that the assumption that the diagnostic software in time-critical applications has enough robustness is valid. When b∈(0,1), the fault information in the current collected signal may not be sufficient for a diagnostic procedure to form a response. If the software responds in the current diagnostic procedure, it means that the robustness of the software is too low, which is inconsistent with the assumption that the diagnostic software in time-critical applications has enough robustness. For this reason, the fault diagnostic system should respond in the next diagnostic procedure (as is shown in Figure 1B). Based on the analysis above, the time lag Δt can be estimated by $\Delta t\approx \left(1+b\right)t_{A}+t_{B}=\left(1+b\right)\frac{n}{f}+O\left[C\left(n\right)\right]$, where b∈(0,1).

## 3. Materials and Methods

The method adopted in this study involves the following steps: Building the optimization design model, narrowing the solution space through prior knowledge, and solving the model. 

### 3.1. Building the Optimization Design Model

In the parametric design, a fault diagnostic software is often defined as a tuple (C,n,f, σ), where σ is the required accuracy of the designed fault diagnostic software. The required accuracy σ is often given in the design document. The diagnostic algorithm C is restricted to the classification method in machine learning. The specific type of the classification method should be determined based on the dataset and the premise of the classification method. The determination of the sampling rate f belongs to the work of the sensor selection before the parametric design. Therefore, the goal of designing the optimal quick diagnostic software can be defined as:

(1) $$\arg\min_{n\in \mathbb{N}}\Delta t=\left(1+b\right)\times \frac{n}{f}+O\left[C\left(n\right)\right];\;b\in \left(0,1\right)$$

In the context of machine learning, the diagnostic algorithm C is a classifier which maps the signal (or time series) $s\in {ℛ}^{n}$ to the system state ℓ∈ℛ. It is learned by a learning algorithm A from a dataset D. The dataset D consists of many signal samples $s\in {ℛ}^{n}$, and the system state ℓ of each sample is known. According to this definition, the diagnostic algorithm C should be represented as $C^{\left(n\right)}$. Subsequently, the classifier is used to classify the new signal sample in order to diagnose faults. 

To guarantee the accuracy of the designed diagnostic software to be no less than the required accuracy σ with the consideration of the effect caused by the design objective, the constraint is built into Equation (3). The accuracy of the designed diagnostic software $\mathrm{acc}\left(C^{\left(n\right)}\right)$ is estimated by the strategy of model selection in machine learning. At present, there are mainly three methods to estimate the accuracy: Holdout, cross validation, and bootstrapping [29]. The design method adopts the holdout approach in this paper. This method sets aside part of the dataset D for learning, and this part is called the training set. The remaining part of the dataset D is called the test set. It is used to provide an unbiased evaluation of the accuracy $\mathrm{acc}\left(C^{\left(n\right)}\right)$, as expressed in Equation (2). N in Equation (2) is the number of signal samples in the test set. In general, the holdout method randomly splits the dataset D into the training set and the test set according to a specified proportion, and the common proportions of the training set and the test set are 8:2 or 7:3.

(2) $$\mathrm{acc}\left(C^{\left(n\right)}\right)=\frac{1}{N}\sum_{n=1}^{N}\left[\left[C^{\left(n\right)}\left(s\right)=\ell \right]\right]$$

(3) $$s.t.\;\mathrm{acc}\left(C^{\left(n\right)}\right)\geq \sigma$$

Combining Equations (1) and (3), the problem in the design of the optimal quick diagnostic system can be formulated as Equation (4). It minimizes the response time Δt and ensures that the accuracy of the designed diagnostic software $\mathrm{acc}\left(C^{n}\right)$ is no less than σ through finding the optimal parameter n. In Equation (4), the parameters b and f are positive numbers, and the time complexity O(·) is a monotonically increasing function no matter which type of algorithm is employed. Therefore, the objective function is also a monotonically increasing function, and it is equivalent to find the minimal n. Based on this, the optimization model can be equivalently expressed as Equation (5).

(4) $$\arg\min_{n\in \mathbb{N}}\Delta t=\left(1+b\right)\times \frac{n}{f}+O\left[C^{\left(n\right)}\left(n\right)\right];\;b\in \left(0,1\right)\;s.t.\;\mathrm{acc}\left(C^{\left(n\right)}\right)\geq \sigma$$

(5) $$\arg\min_{n\in {\mathbb{N}}^{*}}n\;s.t.\;\mathrm{acc}\left(C^{\left(n\right)}\right)\geq \sigma$$

### 3.2. Narrowing the Solution Space Using a Priori Knowledge

The set of natural numbers ℕ is a huge range for solving Equation 5. To improve the efficiency of the procedure, the solution space is narrowed with the help of a priori knowledge about the sampling number n. The sampling number n determines the size of the collected signal. The larger the size of the collected signal, the more information that is available for the diagnostic algorithm. At present, the sampling numbers are 1024, 2048, and 4096 for the signal processing method, which are successfully used for fault diagnosis. Consequently, methods in machine learning also apply these sampling numbers to diagnose and achieve high accuracy [30,31,32]. Obviously, signals of these sizes provide enough information to acquire a satisfactory level of accuracy. Therefore, all these research works actually provide a rational upper bound m for the sampling number, if the required accuracy σ is lower than the accuracy related to the upper bound m. On this basis, an optimal parameter n was determined to be in a new smaller range of sampling numbers. Thus, the objective changed from designing a global optimal quick diagnostic system into designing a local one without any design specification violations, as expressed by Equation (6).

(6) $$\arg\min_{n\in \left[0,m\right]}n\;s.t.\;\mathrm{acc}\left(C^{\left(n\right)}\right)\geq \sigma$$

### 3.3. Solving the Model

Theoretically, a signal of the length m is defined as $s=\left\{\left(t_{i},\;x_{i}\right),i=1,\cdots ,m\right\}$. The timestamp $t_{i}$ is actually a relative time point, and it can generally be ignored. Equation (6) aims to determine a diagnostic method $C^{\left(n\right)}:R^{n}\to R^{1}$ which applies the shortest possible prefix of the signal s, denoted by $s\left[1,n\right]=\{\left(t_{i},\;x_{i}\right)|i=1,\cdots ,n;n<m\}$, to reach the required accuracy σ. To achieve the goal, an improved wrapper method is proposed. This method is based on the wrapper method, one of the three feature selection methods (filter [33,34], wrapper [33,35], and embedding [33,36]). The wrapper method has three basic steps [33,37,38], which are detailed as follows.

(1) A generation procedure to generate the candidate subset of features: The timestamp $t_{i}$ is called the feature of a signal, and the value $x_{i}$ is called the feature value. This step generates a subset of features $G\subset \{t_{i}|i=1,\cdots ,m\}$. The number of elements in G is denoted by k, which is less than m. Then, beside the feature values related to the subset of features G, all the other feature values of each sample in the dataset D are removed to generate a new dataset $D^{*}$.

(2) An evaluation procedure to evaluate the subset: The classifier $C^{\left(k\right)}$ is learned based on the new dataset $D^{*}$. The accuracy of the classifier $C^{\left(k\right)}$ is estimated, which is denoted by $\mathrm{acc}\left(C^{\left(k\right)}\right)$.

(3) A judgment procedure to judge the stopping criterion: Steps 1 and 2 are repeated until the maximal $\mathrm{acc}\left(C^{\left(k\right)}\right)$ is acquired.

In order to solve the optimization problem in this paper, the wrapper method is improved, which is called Algorithm 1. Upon increasing of the sampling number from 1 to n, the subset of features is generated through the extraction of the first n elements in the feature set $\left\{t_{i}|i=1,\cdots ,m\right\}$ in the generation procedure. In the evaluation procedure, the holdout method is applied to evaluate the accuracy of the classifier $C^{\left(n\right)}$. The stopping criterion is that the accuracy $\mathrm{acc}\left(C^{\left(n\right)}\right)$ must be no less than the required accuracy σ. When the criterion is met, the optimal sampling number n is obtained. The tuple (C,n,f, σ) is determined, thus the design is completed. As mentioned previously, the precondition that the required design accuracy σ be lower than the accuracy related to the upper bound m should be satisfied. If not, the solution may not be obtained.

**Algorithm 1** Algorithm of the optimal solution**Step 1.** Determine the dataset D, the classifier h, the learning algorithm A, the required accuracy σ, the proportion τ in the holdout method.**Step 2.** Initialize n=0, $\mathrm{acc}\left(C^{\left(0\right)}\right)$ = 0, $D^{*}=\emptyset$;**Step 3.** Execute the iteration below:     **While**
$\mathrm{acc}\left(C^{\left(n\right)}\right)<\sigma$:       **If**
n<m:       n=n+1;       **For** every sample s
**in**
D:         take s[1,n] into $D^{*}$       **End**       Learn the classifier $C^{\left(n\right)}$ using the learning algorithm A on the dataset $D^{*}$;       Estimate $\mathrm{acc}\left(C^{\left(n\right)}\right)$ using the holdout method;       $D^{*}=\emptyset$       **Else:**     **End while****Step 4.** Output the optimal sampling number n

## 4. Experiment

### 4.1. Experimental Dataset

This paper adopts a dataset of bearing vibration signals from the Case Western Reserve University (CWRU) bearing test data center, and the conditions of data acquisition are given in [39]. The signal samples with single point faults in the inner race way were chosen for the experiments. The fault diameters in the signal samples included 0.007 in, 0.014 in, 0.021 in, and 0.028 in. The sampling frequency f was 12,000 samples/s and the length of each signal sample was at least 480,000. 

According to previous research on bearing fault diagnosis based on the CWRU dataset, the signal length of 4096 was used for fault diagnosis [40]. Therefore, the upper bound m was 4096. According to prior knowledge about the signal length, each original signal sample was split into many segments; each segment was a new signal sample with a length of 4096. All the new signal samples composed the dataset D, and each sample in dataset D had the same label as the original signal from which it was split. The label information is shown in Table 1.

### 4.2. Classifier

**Figure 2:**
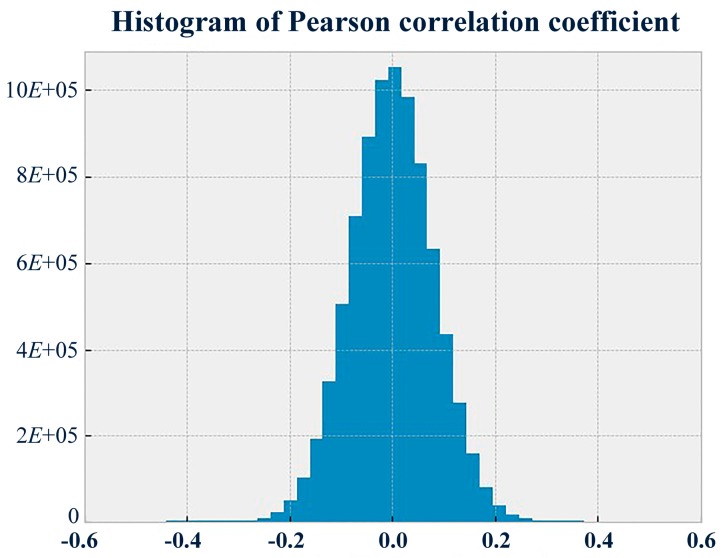
Histogram of Pearson correlation coefficients.

The Naïve Bayes classifier was selected as the diagnostic method C because the dataset D met the model requirement, i.e., the conditional independence assumptions [41]. In machine learning, the Naïve Bayes classifier is a simple probabilistic classifier based on applying Bayes’ theorem with independence assumptions between the features. With the results of the Pearson correlation test at every two timestamps (as is shown in Figure 2), the proportion of the coefficients between −0.3 and 0.3 reached up to 99.75%. The level of coefficients between −0.3 and 0.3 indicated that there was approximately no correlation between every two features. This suggested that the dataset D met the assumptions of the Naïve Bayes classifier.

### 4.3. Experimental Method

Two experiments, denoted as experimental groups A and B, were conducted. In group A, eight diagnostic software were designed based on the proposed method, and the required accuracies σ were 0.6, 0.65, 0.7, 0.75, 0.8, 0.85, 0.9, and 0.95, respectively. Algorithm 1 was executed 200 times, taking into consideration the effect caused by the uncertainty of the training set on accuracy. In group B, the new datasets, shown in Table 2, were composed of signals from dataset D. Based on each new dataset, four diagnostic systems were designed under the required accuracies σ of 0.92, 0.94, 0.96, and 0.98. The algorithm was also executed 200 times due to the same consideration.

## 5. Results and Discussion

In order to present the results intuitively, the values of n derived from the 200 executions of the algorithm were statistically analyzed. Four statistics, including the mean, standard deviation, maximum, and minimum, were chosen to quantify the distributions of the sampling number n, and the histograms were also produced.

### 5.1. Results in Group A

**Figure 3:**
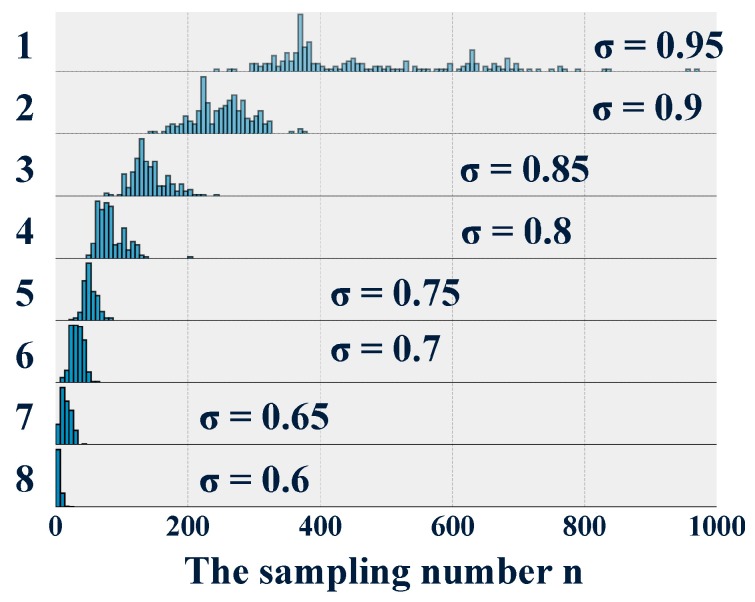
The overlapping densities plot of group A.

In group A, the 200 executions of the algorithm with different accuracy requirements all obtained the optimal sampling number n. Therefore, the parametric design method proved to be feasible to design the optimal quick diagnostic software. The histograms of all eight distributions of sampling number n are presented in Figure 3 using a style of overlapping densities. Obviously, the sampling number n increased with the increasing required accuracy σ. This was consistent with the conclusion that the fault diagnostic system can become more accurate as more signal information becomes available. However, as shown in Table 3, the standard deviation increased from 2.88 to 245.74. In statistics, a high standard deviation indicates that the data points are spread out over a wider range of values. For example, this phenomenon is observed in the histogram with the required accuracy of 0.95. Although the maximal sampling number n can be used to design the diagnostic software conservatively, it is impossible to ignore the accuracy requirement σ causes the designed diagnostic software to become less robust under conditions of noise and uncertainty. In fact, the designed fault diagnostic software needs more signal information due to the increasing accuracy requirement. However, as more signal information becomes available, the noise also increases. Yet, according to the same proportion τ, the training data is generated randomly through the holdout method. This results in the significant uncertainty of the training signal samples. As is shown in Figure 3 and Table 3, with the same increment in the required accuracy σ, the increasing rate of the standard deviation was much higher. This indicated that the fault diagnostic system, designed to reach high accuracy, was very sensitive to noise and uncertainty. Thus, it follows that a high required accuracy reduces the robustness of the designed software.

### 5.2. Results in Group B

**Figure 4:**
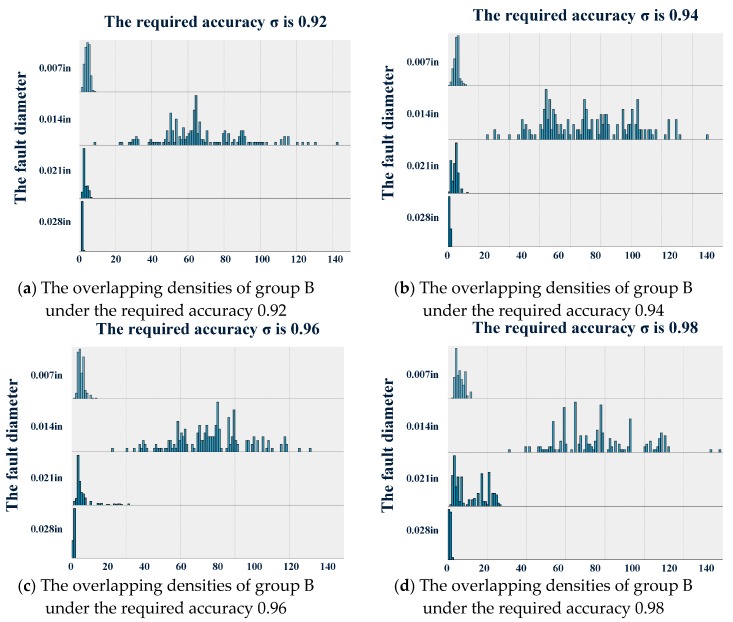
The overlapping densities plot of group B.

For group B, the overlapping densities plot is presented in Figure 4 by combining the designed diagnostic software with the same required accuracy σ in different datasets. The statistics are shown in Table 4, Table 5, Table 6 and Table 7. It can be observed that the means decreased with the increasing fault diameter on the whole. However, it cannot be concluded that the more serious the fault is, the quicker the fault diagnostic software responds. This conclusion was not consistent with the understanding that bearings with a larger fault diameter are often easy to diagnose quickly. Equivalently, as shown in Figure 4, the maximum of the sampling number n in the design based on the dataset $D_{01}$ was less than the minimum sampling number n in the design based on the dataset $D_{02}$. This also means that the response of the diagnostic software to a serious fault is slower than the response to a slight fault. However, it can still be observed that the standard deviation increased with the change in the required accuracy. At the same time, we cannot give a rational explanation for the significant large standard deviation in the design based on the signal samples with a fault diameter of 0.014 in.

### 5.3. Comparison with Related Works

In order to evaluate the proposed design method objectively, it was compared with other methods. The methods used for comparison should provide the estimation of the accuracy, and the sampling number and sampling frequency should be known. Based on this, four methods were chosen, as listed in the first column of Table 8. The comparison was based on the dataset D with the sampling frequency of 12,000 samples/second. The accuracy was estimated based on the dataset D by our calculations, which are listed in the fifth column of Table 8. The sampling number that enabled the generation of a diagnostic result is listed in the third column of Table 8, outside the parentheses. It is worth noting, however, that the listed accuracy may not reflect the real performance of those methods, although the listed accuracy is the best of those obtained after many executions of the proposed algorithm. Based on those accuracies, four diagnostic software were designed. The upper bound of the sampling number was also set to be the same as the sampling number used in the compared methods. In each software design, Algorithm 1 was executed 200 times, taking into consideration the effect caused by uncertainty. The mean of the sampling number n is listed in the third column of Table 8, inside the parentheses. Because the execution time of the diagnostic algorithm $t_{B}$ is determined by the sampling number n and the algorithm type C, the time complexity of each compared method is also provided in Table 8, in the fourth column outside the parentheses (the relationship with the time complexity of our approach is recorded inside the parentheses). According to the comparison of the sampling number and the time complexity, it is reasonable to believe that the designed fault diagnostic software has a smaller time lag Δt than those that were designed by the compared methods under the same accuracy and sampling frequency. Therefore, our approach exhibited superiority in the trade-off between accuracy and the response time.

## 6. Conclusions

This study proposed a parametric design method for an optimal quick diagnostic system. Using this method, an optimal quick diagnostic system can be obtained, which has the smallest response time and a pre-defined accuracy. By means of experimental verification, the feasibility of the parametric design method was validated. Contrary to other existing methods, the proposed approach fulfills the following three characteristics:

First, the measure of the response time was proposed in this method, which provides a common standard to quantitatively assess the improvement of the response time.

Secondly, using the parametric design method proposed in this paper, the designed fault diagnostic software exhibited an obvious improvement in the response time to faults as compared with the response times of traditional methods with the same vibration data. 

Thirdly, the parametric design method exhibited superiority in the trade-off between the accuracy and the response time.

Additionally, the experimental results revealed that the higher the required accuracy is, the less robust the designed diagnostic software is to noise and uncertainty. At the same time, the experimental results demonstrate that serious faults are guaranteed to be diagnosed more quickly by the diagnostic software; although, the more serious the fault is, the more easily the feature will be recognized.

## Figures and Tables

**Table 1:** The information of labels in dataset D.

Label 1	Description
0	This signal label indicates that that there is no fault.
1	This signal label indicates that there is a fault with a diameter of 0.007 in
2	This signal label indicates that there is a fault with a diameter of 0.014 in
3	This signal label indicates that there is a fault with a diameter of 0.021 in
4	This signal label indicates that there is a fault with a diameter of 0.028 in

**Table 2:** The new datasets in group B.

Dataset	Description
$D_{01}$	It includes the signal samples with a fault diameter of 0.007 in and normal signals.
$D_{02}$	It includes the signal samples with a fault diameter of 0.014 in and normal signals.
$D_{03}$	It includes the signal samples with a fault diameter of 0.021 in and normal signals.
$D_{04}$	It includes the signal samples with a fault diameter of 0.028 in and normal signals.

**Table 3:** The statistics for the designed diagnostic system in group A.

Designed Diagnostic System	Required Accuracy σ (%)	Mean	Standard Deviation	Maximum	Minimum
1	0.6	5	2.88	20	2
2	0.65	15.25	7.53	41	3
3	0.7	32.28	9.15	61	8
4	0.75	51.97	9.79	83	26
5	0.8	82.95	20.77	206	50
6	0.85	142.03	27.72	240	79
7	0.9	252.04	43.01	378	144
8	0.95	506.79	245.74	902	240

**Table 4:** The standard deviation of the sampling number in group B.

Required Accuracy σ (%)	Fault Diameter			
	0.007 in	0.014 in	0.021 in	0.028 in
**0.92**	1.33	22.27	1.13	0.12
**0.94**	1.71	28.61	1.77	0.44
**0.96**	2.94	33.03	9.47	0.48
**0.98**	5.40	49.06	20.10	0.76

**Table 5:** The mean of the sampling number in group B.

Required Accuracy σ (%)	Fault Diameter			
	0.007 in	0.014 in	0.021 in	0.028 in
**0.92**	3.87	66.84	2.64	1.015
**0.94**	5.3	91.42	4.305	1.265
**0.96**	8.495	127.585	10.075	1.77
**0.98**	14.805	190.625	28.43	2.64

**Table 6:** The maximum of the sampling number in group B.

Required Accuracy σ (%)	Fault Diameter			
	0.007 in	0.014 in	0.021 in	0.028 in
**0.92**	8	142	6	2
**0.94**	11	170	12	2
**0.96**	22	219	53	3
**0.98**	30	346	66	6

**Table 7:** The minimum of the sampling number in group B.

Required Accuracy σ (%)	Fault Diameter			
	0.007 in	0.014 in	0.021 in	0.028 in
**0.92**	1	8	1	1
**0.94**	1	26	1	1
**0.96**	3	37	2	1
**0.98**	5	78	3	2

**Table 8:** Comparison with related works.

Method	f	n	Time Complexity	σ
W. Du et al. [42]	12 kHz	2048 (>411)	O(nlogn) (>O(n))	88.3%
X. Jin et al. [43]	12 kHz	12,000 (>601)	$O\left(n^{3}\right)$ (>O(n))	93.1%
M. Amar et al. [20]	12 kHz	4104 (>1188)	O(n) (=O(n))	96.2%
X. Zhang et al. [44]	12 kHz	2400 (>1248)	O(n!) (>O(n))	97.3%
